# Is there an increased revision rate due to early tibial component loosening with a modern total knee arthroplasty design? A retrospective analysis from a large volume arthroplasty centre

**DOI:** 10.1186/s42836-024-00264-0

**Published:** 2024-08-03

**Authors:** Bernard H. van Duren, Jonathan France, Reshid Berber, Hosam E. Matar, Peter J. James, Benjamin V. Bloch

**Affiliations:** 1 Nottingham Elective Orthopaedic Services, Nottingham University Hospitals NHS Trust, Nottingham, NG5 1PB UK; 2 Leeds Orthopaedic and Trauma Sciences, Leeds Institute of Rheumatic and Musculoskeletal Medicine, University of Leeds, Leeds, LS2 9JT UK; 3 University of Nottingham, School of Medicine, Nottingham, UK

**Keywords:** Total knee arthroplasty, Loosening, Revision, Survival

## Abstract

**Background:**

The Attune TKR was introduced in 2011 as a successor to its predicate design The PFC Sigma. However, following reports of early failures, there are ongoing concerns related to increased loosening rates. Given the concerns, this study aimed to compare revision rates of the Attune implant to an established predicate, and other implant designs used in a high-volume arthroplasty center.

**Methods:**

We identified 10,202 patients who underwent primary cemented TKR at our institution with a minimum of 1 year follow-up, involving 2406 Attune TKR (557 S +), 4642 PFC TKR, 3154 other designs. Primary outcomes were revision for all-causes, aseptic loosening of any component, and aseptic tibial loosening. Kaplan–Meier survival and Cox regression models were used to compare groups. Matched cohorts were selected for radiographic analysis.

**Results:**

308 knees were revised. The Attune cohort had the lowest risk of revision, with a rate of 2.98 per 1000 implant-years while the PFC and All Other Implant groups had a rate of 3.15 and 4.4 respectively. Aseptic loosing was the most common cause for revision, with 76% (65/88) involving the tibia. Survival analysis showed no significant differences between the Attune and other cohorts. Radiolucent lines were detected in 7.1% of the Attune S + group, 6.8% of the standard Attune group, and 6.3% of the PFC group, with no significant differences found between them.

**Conclusion:**

This study represents the largest non-registry review of the Attune TKR in comparison to a predicate and other designs. There was no significant increased revision rate for all-cause revision or aseptic loosening, or peri-implant radiolucencies. It appears that increased loosening may not be as concerning as originally thought.

**Level of Evidence:**

Level III.

## Introduction

Total knee arthroplasty (TKA) is a successful operation for end-stage arthritis of the knee and has been proven to ease pain and improve function. Excellent long-term implant survival has been reported by national registries [1, 2]. The trend in TKA is projected to increase over the next few decades [3]. Despite this, up to 20% of patients remain dissatisfied following TKA [4, 5]. A proportion of TKAs will need early revision, with the most common reasons for early failure being infection, aseptic loosening, and instability [4]. Around a third of revisions will occur within the first two years [5].

Aseptic loosening is the most reported reason for revision in the UK’s National Joint Registry (NJR), with 1.16 revisions per 1000 implant-years being reported across all knee arthroplasties [1]. The reasons for loosening are dependent on a combination of surgical technique and implant design resulting in increased relative motion at the implant interfaces. Design features, such as implant geometry (stem length, keel shape, cement pockets) and surface texture, have been reported to influence implant interface stability [6–11].

A novel TKA design was introduced in 2011. The new design has an increased conformity between the femoral component and polyethylene insert, optimized patellofemoral conformity and mechanics, an improved polyethylene locking mechanism for fixed bearings and an increased range of sizes for diverse populations in comparison to its predicate design [12]. However, following reports of early failures due to aseptic loosening of the tibial component [8, 13–16], there have been ongoing concerns about increased loosening rates with this knee system. In 2017, a redesigned tibial baseplate was introduced, which included an undercut cement pocket area and an increased surface roughness (3.0–6.5 Ra) to enhance cement bonding [13].

This novel TKA system was introduced to our unit in December 2011 and has since become the primarily used knee system. Prior to this, a predicate design with an excellent long-term track record was routinely used and is still used by some surgeons. Other designs have also been used within our center. Given the reports regarding early tibial loosening, the aim of this study was to evaluate the overall revision rates and those specific to aseptic loosening of the novel implant design in comparison to an established predicate as well as to all other implant designs used in this high-volume arthroplasty center. Additionally, a radiographic analysis was undertaken to establish the presence of radiolucent lines.

## Methods

### Patients

This retrospective consecutive study included all patients who underwent primary cemented TKA at our institution between 1st April 2003 and 31st March 2022 with a minimum of 1 year follow-up. Institutional approval for this study was obtained. We identified our cohort through local prospective electronic databases and linkable data obtained from the NJR. We identified patients’ age, gender, American Society of Anaesthesiologists (ASA) score, and indication for surgery. Revisions and re-operations were identified using a combination of our own database, a review of clinical notes, and where necessary, contacting the patient or, if they had died, the patient’s general practitioner.

### Demographics

**Fig. 1:**
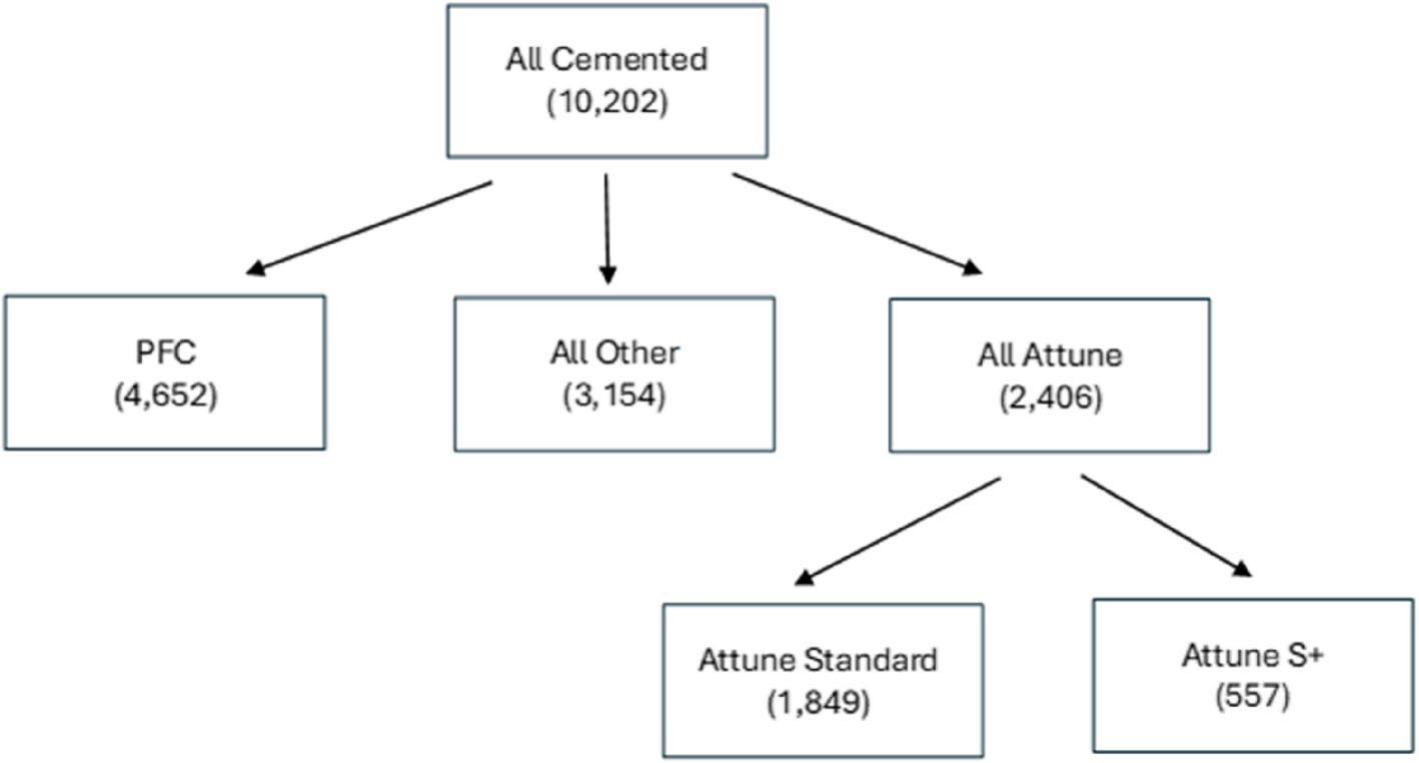
Flowchart to illustrate the subdivision of the implant groups

A total of 10,202 patients across all the cohorts were included (Fig. 1). An overview of the cohort distributions and demographics is given in Table 1. Mean follow-up was 5.3, 9.1, and 9.9 years for the Attune, PFC, and all other implant groups respectively. There were no significant differences in the age, gender, primary indication, and ASA distributions between the cohorts.

**Table 1:** Overview of patient cohorts and associated demographics. *SD* standard deviation, *IQR* inter-quartile range

	**All Cemented**	**All Attune**	**All Other Cemented**	**PFC**	**Other Cemented**	**Attune Standard**	**Attune S + **
**Total No. (BMI > 30)**	10202	2406	7796	4642	3154	1849	557
**Total Prosthesis Years**	86,098	12,731	73,367	42,200	31,167	11,134	1598
**Mean Follow-Up (SD) {years}**	8.4 (4.4)	5.3 (2.3)	9.4 (4.4)	9.1 (4.3)	9.88 (4.6)	6.0 (2.1)	2.9 (1.0)
**Range {years}**	1–20.1	1–11.2	1–20.1	1–19.9	1–20.1	1–11.2	1–4.6
**Median (IQR) {years}**	7.8 (4.9–11.5)	5.2 (3.5–6.9)	9.2 (6.0–12.7)	9.0 (5.8–12.1)	9.5 (6.2–13.4)	6.0 (4.6–7.6)	3.2 (1.9–3.7)
**Female**	5922 (58%)	1463 (61%)	4459 (57%)	2681 (58%)	1778 (56%)	1135 (61%)	328 (59%)
**Age {years}**							
< 65	2933 (29%)	694 (29%)	2239 (29%)	1418 (31%)	821 (26%)	553 (30%)	141 (25%)
65–74	3497 (34%)	889 (37%)	2883 (37%)	1668 (36%)	1215 (39%)	683 (37%)	206 (37%)
≥ 75	3772 (37%)	823 (34%)	2674 (34%)	1556 (36%)	1118 (35%)	613 (33%)	210 (38%)
**ASA**							
I	891 (9%)	143 (6%)	748 (10%)	354 (8%)	394 (12%)	127 (7%)	16 (3%)
II	7116 (70%)	1703 (71%)	5413 (69%)	3311 (71%)	2102 (667%)	1318 (71%)	385 (69%)
III	2148 (21%)	552 (23%)	1596 (20%)	957 (21%)	639 (20%)	397 (21%)	155 (28%)
IV	45 (< 1%)	8 (< 1%)	37 (< 1%)	18 (< 1%)	19 (< 1%)	7 (< 1%)	1 (< 1%)
V	2 (< 1%)	0 (< 1%)	2 (< 1%)	2 (< 1%)	0 (< 1%)	0 (0%)	0 (0%)
**No Surgeons**	32	15	27	24	19	13	11
**Indication**							
OA	9955 (98%)	2349 (98%)	7606 (98%)	4517 (97%)	3089 (98%)	1808 (98%)	541 (97%)
Inflammatory Arthropathy	187 (2%)	42 (2%)	145 (2%)	95 (2%)	50 (2%)	31 (2%)	11 (2%)
AVN	16 (< 1%)	3 (< 1%)	13 (< 1%)	10 (< 1%)	3 (< 1%)	1 (< 1%)	2 (< 1%)
Trauma	15 (< 1%)	8 (< 1%)	7 (< 1%)	5 (< 1%)	2 (< 1%)	6 (< 1%)	2 (< 1%)
Prev Infection	4 (< 1%)	1 (< 1%)	3 (< 1%)	3 (< 1%)	0 (< 1%)	1 (< 1%)	0 (0%)
Other	23 (< 1%)	3 (< 1%)	20 (< 1%)	11 (< 1%)	10 (< 1%)	2 (< 1%)	1 (< 1%)
**Meniscal Constraint**							
Unconstrained Mobile	1608 (16%)	1498 (62%)	110 (2%)	8 (< 1%)	102 (3%)	975 (53%)	523 (94%)
Unconstrained Fixed	6557 (64%)	666 (28%)	5891 (75%)	3158 (68%)	2733 (87%)	666 (36%)	0 (0%)
PS mobile	978 (10%)	191 (8%)	787 (10%)	659 (14%)	128 (4%)	157 (8%)	34 (6%)
PS Fixed	1059 (10%)	51 (2%)	1008 (13%)	817 (18%)	191 (6%)	51 (3%)	0 (0%)

### Implants

The Attune TKA (Depuy Synthes) system was introduced to our unit in December 2011 and has since become the primarily used knee system. Prior to this, a predicate design with an excellent long-term track record (PFC Sigma, DePuy Synthes) was routinely used and continues to be used by some surgeons. Other designs of TKA, including Columbus (B Braun/Aeculap), E-Motion (B Braun/Aesculap, ACS (Implantcast), Brmingham Knee Replacement (JointMedica), Advance MP (MicroPort), Genesis II (Smith & Nephew), Journey (Smith & Nephew), Kinemax (Stryker), Scorpio (Stryker), Triathlon (Stryker), Nexgen (Zimmer), Vanguard (Zimmer), have also been used within our center.

### Cementing technique

All procedures were performed by or under the direct supervision of fellowship-trained arthroplasty surgeons. Prior to cementation, cancellous bone was cleaned of lipid deposits, blood, and bone debris using pulse lavage and dried using a swab under compression till immediately before cement application. A single-stage cementing technique was used by employing either Palacos R + G (Heraeus Medical, Hanau, Germany) or SmartSet GHV (DePuy Synthes, Warsaw, IN, USA) vacuum-mixed cement. Cement was applied to both bone and implant contact surfaces. On application to bone surfaces, care was taken to pressurize cement into the cancellous bone surface. Care was exercised to ensure both cement surfaces were dry and debris-free before the application of the implant. Implants were applied, impacted, and residual cement was removed prior to extending the leg and keeping the leg in extension for ten minutes till the cement had been set.

### Statistical analysis

Continuous descriptive statistics were presented as, where appropriate, means, median values, ranges, and 95% confidence intervals. Where categorical variables were compared, *Pearson chi-square test* was used and *t-test/Mann–Whitney U* tests were utilized for continuous variables.

Kaplan–Meier curves were used to assess the survival of the Attune TKA in comparison to a predicate design (PFC, DePuy Synthes, Warsaw, IN, USA), and all other cemented TKAs. A further comparison was made between Attune using the regular and the newer S + tibial components. The outcome measures assessed were as follows:

- All-cause revision was defined as removal or exchange of the femoral, tibial, insert, or patella components. These included isolated bearing exchange for infection (debridement, antibiotics and implant retention; DAIR) and secondary patellar resurfacing.
- Revision for aseptic loosening was defined as any revision for which the primary cause was recorded as aseptic loosening of any component.
- Revision for aseptic tibial loosening was defined as any revision for which the tibial component was recorded as being loose (excluding infection and fracture).

Cox proportional hazard regression analysis was undertaken to investigate the effects of variables on survival. The variables adjusted were age at surgery, gender, primary indication, ASA score, and meniscal constraint. To assess whether a fitted Cox regression model adequately described the data, the assumption of proportional hazards was investigated by calculation and visual inspection of Schoenfeld residuals. The level of significance was taken to be a *P* < 0.05. RStudio (version 2022.02.2) was used to perform the analyses.

### Radiographic analysis

A radiographic analysis was undertaken to compare the presence of radiolucent lines in the Attune S + , original Attune, and P.F.C. implants. Using a prospectively maintained arthroplasty database, we identified 300 patients receiving the Attune S + TKA, and two additional matched cohorts of patients receiving an original Attune TKA and P.F.C. TKA as the comparator groups. The cohorts were matched 1:1 for duration of follow-up, age, and gender, using the nearest neighbour matching method. Those that had two radiographs available for analysis (immediately postoperatively and 6 weeks or more postoperatively) were included. For the purposes of radiographic analysis, the most recent radiographs were compared to the immediate postoperative radiographs for progressive lucency. The technique described by Meneghini et al*.* [17] for looking for the presence of radiolucent lines was used. The review was carried out by two independent clinicians. Where there was disagreement about the presence of radiolucent lines, a third reviewer was consulted.

## Results

### Revisions

Overall, 308 implants underwent revision (Table 2) equating to 3.58 revisions per 1000 implant-years. The lowest risk of revision was noted in the Attune cohort, with 2.98 per 1000 implant-years whereas the PFC and All Other Implant groups had 3.15 and 4.4 revisions per 1000 implant-years, respectively. Aseptic loosening was the most common cause for revision across all cemented implants, with 76% (65 of 88) of these cases involving loosening of the tibia. Infection (77), instability (36), and pain (26) were the next most reported reasons for revision with progressive OA (22), stiffness (18), periprosthetic fracture (10), malalignment (9), implant wear (5), and subluxation (2), accounting for the remaining cases (Table 2).

**Table 2:** Overview of revision cases and primary indications for revision

	**Total No. Revisions**							**No. Revisions per 1000 Implant years**						
	**All Cemented**	**Attune**	**All Other Cemented**	**PFC**	**Other Cemented**	**Attune Standard**	**Attune S + **	**All Cemented**	**Attune**	**All Other**	**PFC**	**Other Cemented**	**Attune Standard**	**Attune S + **
**Total No. Revisions**	308	38	270	133	137	34	4	3.58	2.98	3.68	3.15	4.40	3.05	2.50
**Pain**	26	0	26	20	6	0	0	0.30	0.00	0.35	0.47	0.19	0.00	0.00
**Infection**	77	6	71	36	35	5	1	0.89	0.47	0.97	0.85	1.12	0.45	0.63
**Aseptic Loosening (All)**	88	9	79	22	57	8	1	1.02	0.71	1.08	0.52	1.83	0.72	0.63
Loosening Tibia	65	7	58	13	45	6	1	0.75	0.55	0.79	0.31	1.44	0.54	0.63
Loosening Femur	45	6	39	6	33	5	1	0.52	0.47	0.53	0.14	1.06	0.45	0.63
Loosening Patella	13	2	11	6	5	5	1	0.15	0.16	0.15	0.14	0.16	0.45	0.63
**Dislocation/Subluxation**	2	0	2	1	1	0	0	0.02	0.00	0.03	0.02	0.03	0.00	0.00
**Periprosthetic Fracture**	10	2	8	6	2	2	0	0.12	0.16	0.11	0.14	0.06	0.18	0.00
**Implant Wear/Fracture**	5	0	5	2	3	0	0	0.06	0.00	0.07	0.05	0.10	0.00	0.00
**Instability**	36	3	33	17	16	2	1	0.42	0.24	0.45	0.40	0.51	0.18	0.63
**Malalignment**	9	1	8	6	2	1	0	0.10	0.08	0.11	0.14	0.06	0.09	0.00
**Stiffness**	18	5	13	5	8	5	0	0.21	0.39	0.18	0.12	0.26	0.45	0.00
**Progressive OA**	22	10	12	8	4	10	0	0.26	0.79	0.16	0.19	0.13	0.90	0.00
**Other**	15	2	13	10	3	1	1	0.17	0.16	0.18	0.24	0.10	0.09	0.63

The overall rate of tibial loosening was 0.75 revisions per 1000 implant-years. The lowest rate of tibial loosening was in the PFC group, with a rate of 0.31 and highest in the all-other implant group, with 1.44 revisions per 1000 implant-years. The overall Attune revision rate for tibial loosening was 0.55 per 1000 implant-years. This was not significantly different to the other groups. There was no significant difference between the standard Attune and S + designs either (Table 2).

### Survival analysis

**Fig. 2:**
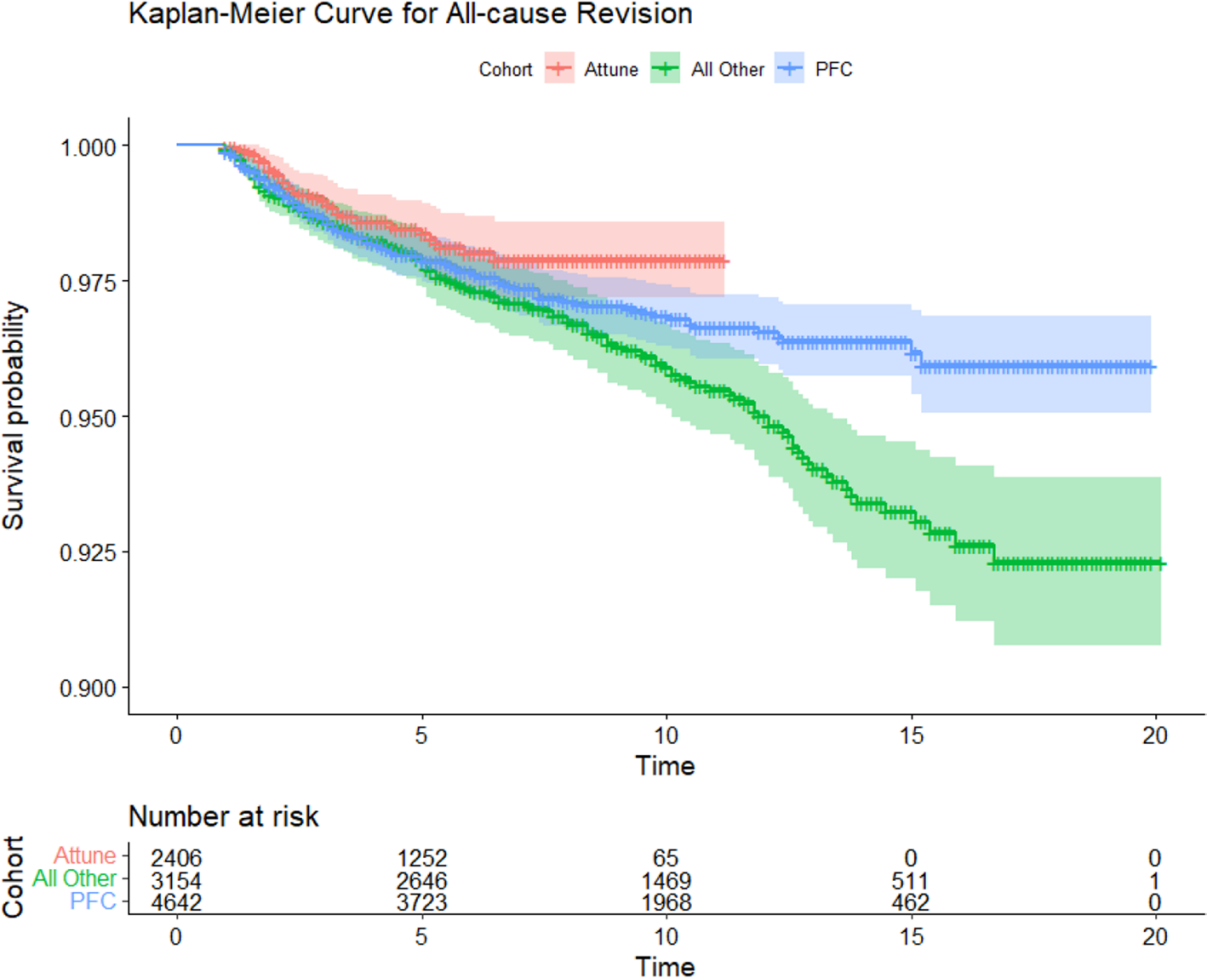
Kaplan–Meier survival curves comparing Attune, PFC, and AOCI cohorts for all-cause revisions. Pairwise comparisons using Log-Rank test: Attune vs. PFC = 0.15, Attune vs. All Other = 0.07, PFC vs. All Other = 0.01

**Fig. 3:**
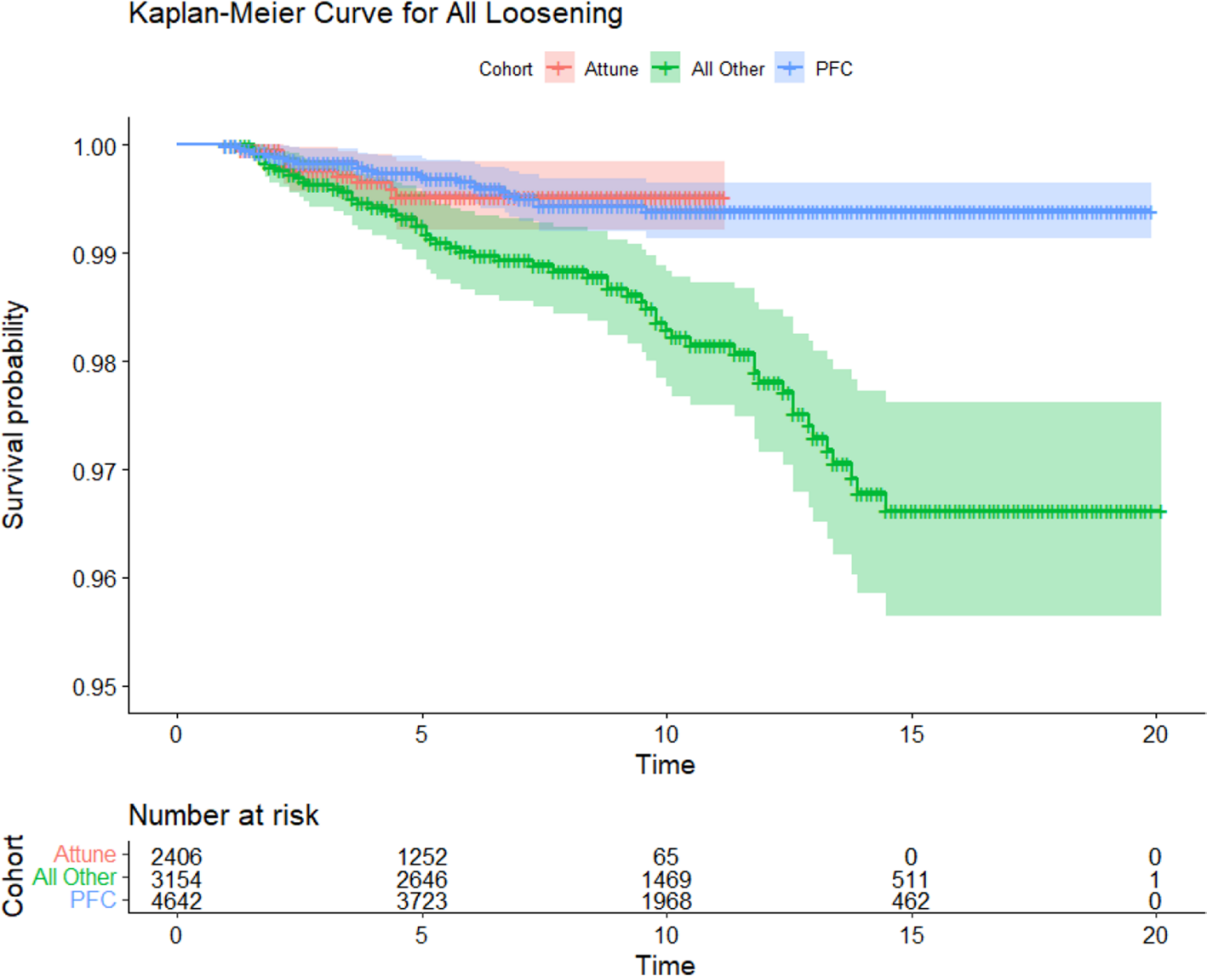
Kaplan–Meier survival curves comparing Attune, PFC, and AOCI cohorts for revision for all component loosening. Pairwise comparisons using Log-Rank test: Attune vs. PFC = 0.77, Attune vs. All Other = 0.06, PFC vs. All Others ≤ 0.001

**Fig. 4:**
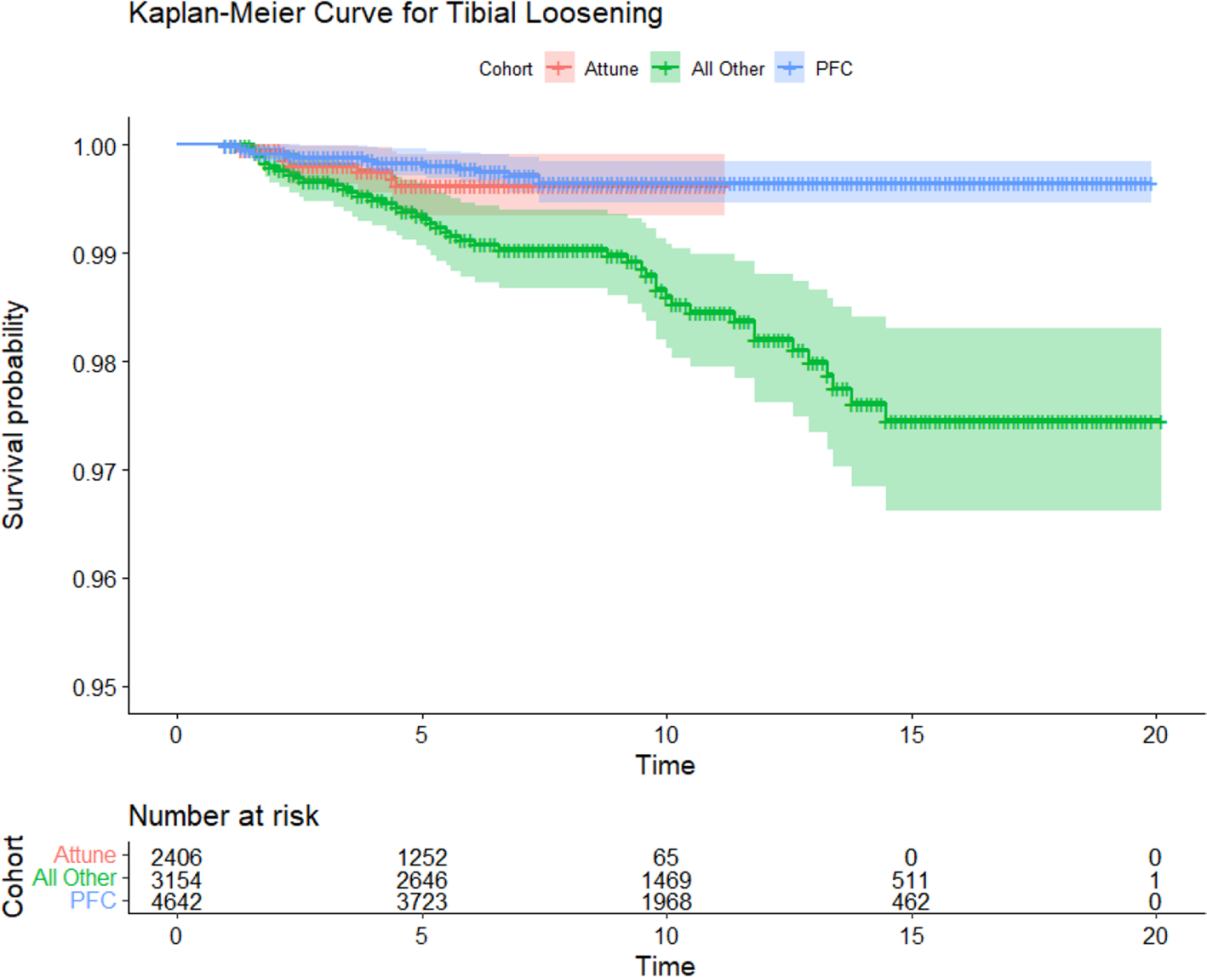
Kaplan–Meier survival curves comparing Attune, PFC, and AOCI cohorts for revisions for tibial component loosening. Pairwise comparisons using Log-Rank test: Attune vs. PFC = 0.47, Attune vs. All Other = 0.05, PFC vs. All Other ≤ 0.001

**Fig. 5:**
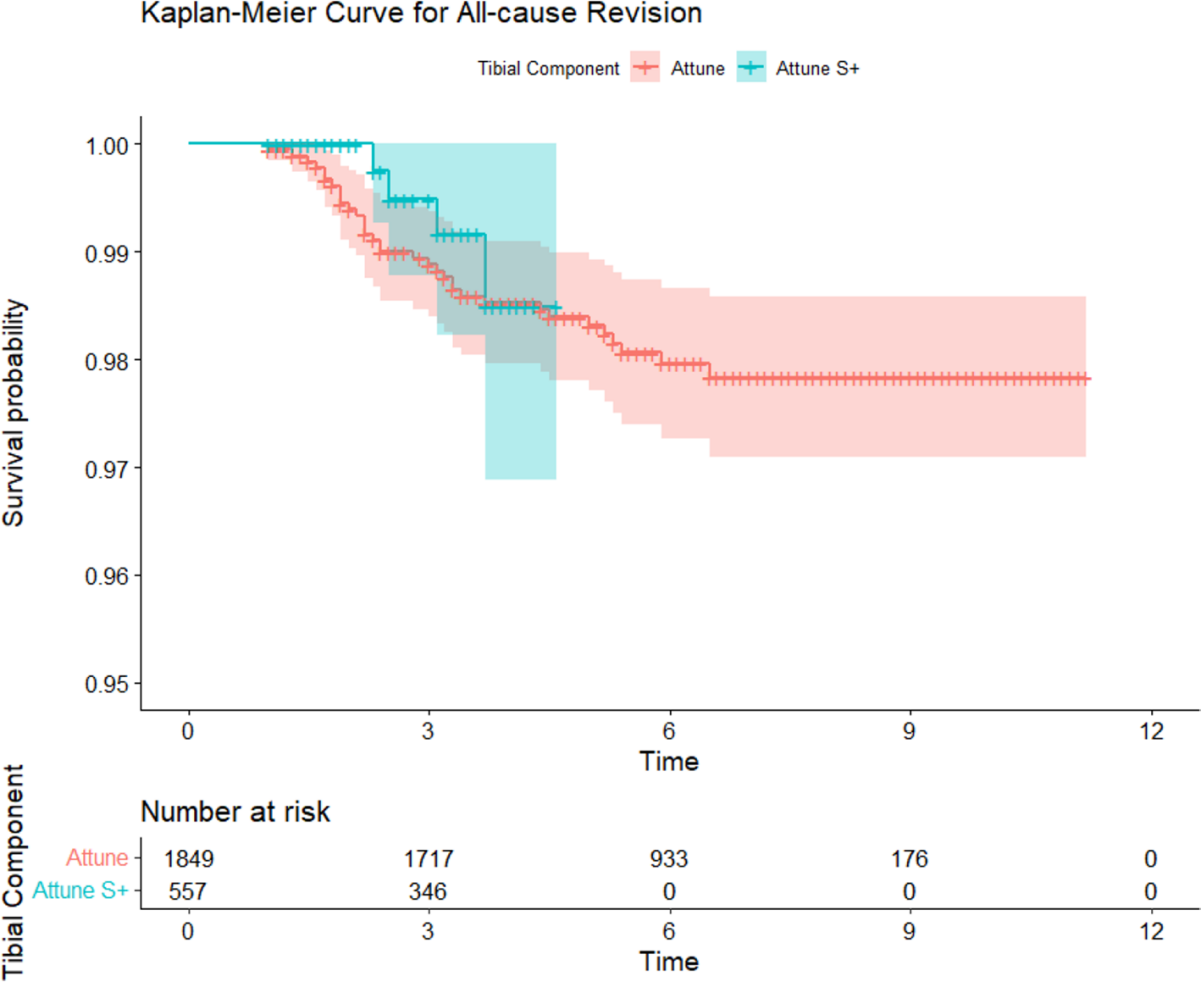
Kaplan–Meier survival curves comparing Attune and S + cohorts for all-cause revisions. Pairwise comparisons using Log-Rank test: Attune vs. Attune S +  = 0.55

**Fig. 6:**
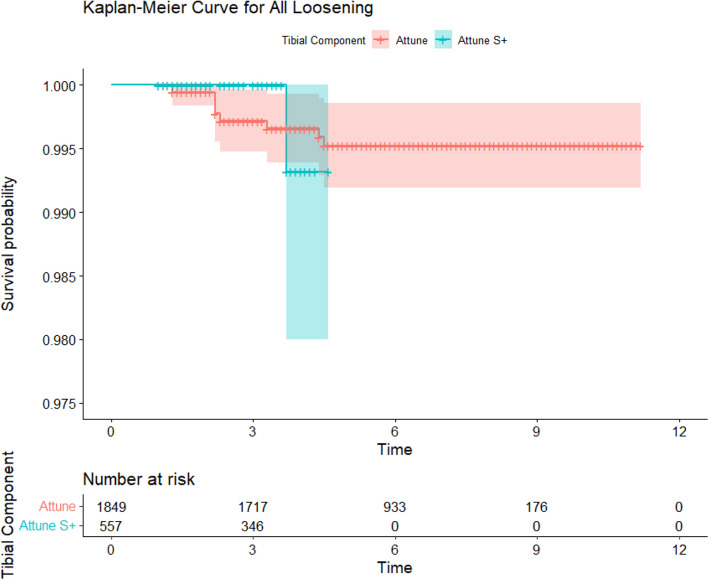
Kaplan–Meier survival curves comparing Attune and S + cohorts for revision for all component loosening. Pairwise comparisons using Log-Rank test: Attune vs. Attune S +  = 0.85

**Fig. 7:**
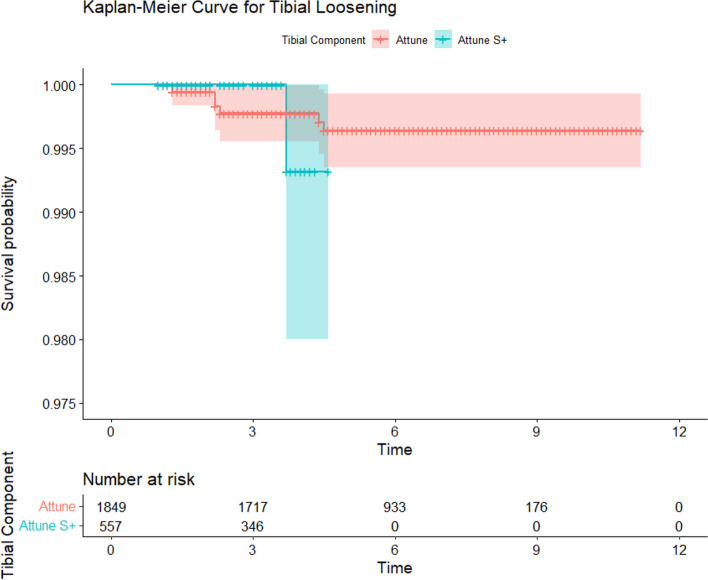
Kaplan–Meier survival curves comparing Attune and S + cohorts for revision for tibial component loosening. Pairwise comparisons using Log-Rank test: Attune vs. Attune S +  = 0.85

Survival analysis comparing the Attune cohort to the PFC and All Other Cemented Implant (AOCI) cohorts showed no significant differences between the Attune and PFC cohorts in all-cause revision, aseptic loosening, or tibial loosening (*P* = 0.15, 0.77, 0.47 respectively) (Figs. 2, 3 and 4). The PFC cohort demonstrated significantly improved survival outcomes for all-cause revision, aseptic loosening, and tibial loosening as compared to the AOCI cohort (*P* = 0.01, < 0.001, < 0.001 respectively). Survival analysis comparing the standard Attune design to the S + cohort (Figs. 5, 6 and 7) showed no significant difference in survival between the two for all-cause revision, revision for aseptic loosening, and tibial loosening (*P* = 0.15, 0.77, 0.47 respectively).

Table 3 gives an overview of the proportional hazard ratios (HR) relevant to reference variables as determined by using Cox regression analysis. A decreasing HR of revision was noted with increasing age, which was significant for all-cause revision and aseptic loosening. There were no significant changes in revision related to gender or primary indication for surgery. The level of constraint provided by the articulation showed an increased HR of 1.8 for all-cause revision in cruciate retaining fixed bearing implants, which was significant (*P* = 0.04). Also a lower HR of 0.07 (*P* = 0.02) and an HR of 0.16 (*P* = 0.04) were noted for aseptic loosening and isolated tibial loosening in the fixed bearing posterior stabilized group.

**Table 3:** The effects of variables upon survival given as proportional hazards calculated using cox regression analysis

	**All Cause Revision**		**Revision for Loosening Any Component**		**Revision for Loosening Tibial Component**	
	HR	*P*-value	HR	*P*-value	HR	*P*-value
**Age**						
< 65	1	-	1	-	1	-
65–74	0.6	< 0.001	0.5	0.01	0.5	0.005
≥ 75	0.4	< 0.001	0.2	< 0.001	0.2	< 0.001
**Gender**						
Female	1	-	1	-	1	-
Male	1.2	ns	1.3	ns	0.9	ns
**Indication**						
OA	1	-	1	-	1	-
Inflammatory Arthropathy	0.9	ns	1.10	ns	7.00 × 10^–1^	ns
AVN	1.7	ns	4.80 × 10^–7^	ns	3.50 × 10^–7^	ns
Trauma	3.6	ns	5.1	ns	7.4	ns
Prev Infection	7.60 × 10^–7^	ns	8.70 × 10^–7^	ns	7.70 × 10^–7^	ns
Other	7.50 × 10^–7^	ns	4.90 × 10^–7^	ns	3.80 × 10^–7^	ns
**ASA**						
I	1	-	1	-	1	-
II	0.7	ns	0.6	0.03	0.6	ns
II	0.9	ns	0.5	0.04	0.4	0.05
IV	0.7	ns	3.20 × 10^–7^	ns	2.40 × 10^–7^	ns
V	1.02 × 10^–6^	ns	1.50 × 10^–6^	ns	1.20 × 10^–6^	ns
**Meniscal Constraint**						
Unconstrained Mobile	1	-	1	-	1	-
Unconstrained Fixed	1.8	0.04	0.6	ns	0.4	ns
PS mobile	1.8	ns	0.6	ns	0.4	ns
PS Fixed	0.8	ns	0.07	0.02	0.16	0.04

*HR* Hazard Ratio, *ns* not significant

### Radiographic analysis

241 patients in the S + group, 218 in the Attune group, and 270 in the PFC group had radiographs available for analysis, with a mean radiographic follow-up of 269, 475, and 387 days, respectively. In the S + group 1, there were 17 patients (7.1%) with tibial RLLs; in the standard Attune group, 15 (6.8%) had tibial RLLs, and in the PFC group, 17 (6.3%) had RLLs. In all the groups, the tibial RLLs were predominantly in zone 1 (67%, 58%, 71% for S + , Attune, and PFC groups, respectively) or 2 (58%, 58%, 64% for S + , Attune, and PFC groups, respectively) on the AP view. These RLLs were all less than 2 mm and were non-progressive. No significant differences were noted between the groups.

## Discussion

This study compared the survival of the Attune TKA to its predicate design, the PFC, and all other TKA designs used in a single institution. Reassuringly, in this study, no evidence of increased aseptic loosening was found with the Attune implant as compared with established TKA systems. Survival analysis showed no significant difference in survival of the Attune knee when compared to the PFC or all other knee arthroplasty designs for all-cause revision, revision for aseptic loosening, and revision for tibial loosening. Similarly, radiographic analysis did not reveal any significant differences in radiolucent line formation of the Attune design when compared to the PFC.

Aseptic loosening is recognized to be multifactorial, with surgeon factors, cementation technique, implant factors, and patient factors playing important roles [18, 19]. Lower volume surgeons have been shown to have higher revision rates, likely related to technical skill [20]. Cementation technique factors included the cement type, cement application methods, and cement thickness [21–23]. Some authors have cited implant design factors, which included component shape and surface roughness [11, 24–27]. Finally, patient factors such as increased BMI and younger age have also been linked to increased rates of aseptic loosening [28–31].

This study, to our knowledge, represents the largest non-registry review of the original Attune Knee to date. In our cohort of 2 406 Attune TKAs, there were 38 (1.6%) revisions for all-causes and 9 (0.4%) revisions for aseptic loosening. Other studies looking at loosening in the Attune implant have reported revision rates between 0%–11.5% for all cause revision and 0%–10.2% for aseptic loosening [12–14, 32–40] (Table 4). Of these studies, seven compared the Attune implant to a control group [12, 14, 33, 34, 36, 37, 39], with only one study [33] having found a significantly higher revision rate for aseptic loosening in the Attune cohort. All the other studies, as was the case in this study, revealed no significant differences. Similarly, registry data reported revision rates that were comparable to established TKA prostheses [1, 2].

**Table 4:** An overview of published studies reporting on all-cause and aseptic loosening revision rates for the Attune implant

	**Attune**						**Control**				
**Study (year)**	**Study type**	**Insert**	**Mean follow-up**	***n***	**All-cause revision**	**Revision for aseptic loosening**	**Implant**	**Mean follow-up**	***n***	**All-cause revision**	**Revision for aseptic loosening**
Willburger & Oberberg (2022) [12]	Prospective cohort	CR	60	30	0 (0%)	0 (0%)	Sigma PFC	60	30	0 (0%)	0 (0%)
Torino et al. (2022) [13]	Retrospective cohort	FB & RP	42	742	18 (2.4%)	10 (1.3%)	-	-	-	-	-
van Loon et al. (2021) [32]	Prospective cohort	RP CR/PS	24	200	1 (0.5%)	0 (0%)	-	-	-	-	-
Lachiewicz et al. (2021) [33]	Retrospective cohort	FB PS	23.7	166	19 (11.5%)	17 (10.2%)	multiple	25	511	13 (2.5%)	2 (0.4%)
Robinson et al. (2021) [34]	Retrospective cohort	FB CR	24	96	0 (0%)	0 (0%)	Sigma PFC *n* = 40, Vanguard *n* = 52	24	92	0 (0%)	0 (0%)
Song et al. (2022) [41]	Retrospective cohort	PS	40.8	500	2 (0.4%)	0 (0%)	-	-	-	-	-
Jin et al. (2020) [36]	Retrospective cohort	FB PS	28.4	68	0 (0%)	0 (0%)	Persona	29.1	74	0 (0%)	0 (0%)
Kaptein et al. (2020) [37]	RCT	FB CR	24	37	0 (0%)	0 (0%)	Sigma PFC	34	37	1 (2.7%)	0 (0%)
Hoskins et al. (2020) [20, 38]	Retrospective cohort	FB/RP, CR/PS	21	122	0 (0%)	0 (0%)	-	-	-	-	-
Bloch et al. (2020) [40]	Retrospective Cohort	FB/RP, CR/PS	2 (min)	500	6 (1.2%)	1 (0.2%)	Sigma PFC	2 (min)	500	11	1 (0.0.2%)
Staats et al. (2019) [39]	Retrospective cohort	FB/RP, CR/PS	19	276	3 (1.1%)	0 (0%)	Sigma PFC	25	253	5 (2.0%)	1 (0.4%)
Ranawat et al. (2017) [14]	Retrospective matched cohort		22.8	100	1 (1%)	0 (0%)	Sigma PFC	24	100	0 (0%)	0 (0%)
Current Study	Retrospective cohort	FB/RP, CR/PS	63.6	2406	38 (1.6%)	9 (0.4%)	PFC	109.2	4642	133 (2.9%)	22 (0.5%)
							Multiple	118.6	3154	137 (4.3%)	57 (1.8%)

Radiolucent lines (RLLs) at the implant-cement and bone-implant interfaces are a radiographic sign associated with component loosening. As they are potential harbingers of loosening, many studies have looked at the occurrence of RLLs with the Attune knee [14, 32–39, 42, 43]. A study reported a wide variation in the incidence of RLLs, ranging from 0% [14, 32] to as high as 66% [33]. Six of these studies compared the occurrence of RLLs in the Attune to control cohorts [14, 34, 36, 37, 39, 42], of which two studies by Kaptein et al. [37] and Staats et al. [39] showed a significantly increased rate of RLLs in their respective Attune cohorts. The other studies [14, 34, 36, 42], as was the case in this study, did not show any significant differences in RLLs between Attune and control cohorts. Furthermore, radiostereometric analyses (RSA) have shown low rates of maximum total point motion at 2 years, which is predictive of a low risk of medium to long-term failure due to aseptic loosening [44].

There is a large variability in the incidence of RLLs among studies, which raised the question of whether analysis of RLLs ares a reliable means of comparing potential loosening [45]. Such a wide variation in RLLs in the literature is likely multi-factorial, involving variation in reviewer interpretation and quality of radiographs (image resolution, whether true AP or lateral views), among others. The assessment of RLLs must follow a set protocol and the radiographic beam should be placed parallel to the implant components [46]. It is difficult to know whether a reproducible technique was used in the previous studies. While attention has been drawn to increased RLLs associated with the Attune design, the clinical significance remains unclear [45]. The presence of RLLs do not specifically imply loosening [45]. The diagnosis of early loosening remains difficult, requiring assessment of both radiological and clinical factors and ultimately intraoperative confirmation at revision.

Cox regression analysis showed HR for all-cause revision, aseptic loosening, and tibial loosening decreased significantly with increasing age. This finding was not unexpected since it could be expected that younger patients are more demanding on their implants [47–49]. It was also noted that decreased HR for aseptic loosening was associated with higher ASA grades (significant for ASA II, III, Table 3). We surmise that the more active patients were more likely to have a lower ASA grade and, therefore, placing increased mechanical demand on their implants. Interestingly, a significantly lower HR was noted for the PS fixed-bearing implants when compared to the other meniscal constraint types (Table 3). No clear reason could explain this finding and we assumed that this might be ascribed to the PS fixed bearing only representing 2% of the overall ATTUNE cohort (Table 1).

This study had some inherent limitations associated with its retrospective design. It was not a randomized controlled trial, and although there were no obvious significant differences in patients’ demographics among the cohorts, it is possible that there were differences that may have affected the revision rate. Multiple surgeons might have different implant preferences and inevitably differences in technique used. The most prevalent difference lay in that the majority of the rotating platform designs were performed by surgeons using a gap balancing approach and most fixed bearing implants was done by employing measured resection. There were also some variations in implant configuration. Previous studies have found very little clinical difference between PS and CR components, other than a potential small increase in range of motion with PCL resection [50–52]. Moreover, the PFC group also had a higher proportion of fixed-bearing prostheses due predominantly to the use of all-polyethylene tibial components. All-polyethylene tibias performed as well as metal-backed modular tibial components [50, 53], and, while mobile-bearing TKA prostheses may potentially improve range of motion and clinical outcome scores, there was no proven difference in implant survivorship [54–57].

The all-other cemented implant cohort contained multiple different implant designs, meaning that each implant design was not evaluated individually. Individual comparison of other implants was beyond the scope of this study, which aimed to assess the risk of loosening in the Attune design when compared to other implants used within our unit. As such, we could not draw any conclusions about other designs but rather could only establish that the Attune design overall did not show a significant increase in all-cause revision nor revision for loosening, either of any or of isolated tibial component in the short- to mid-term. This was a reassuring finding but continued vigilance and review of our outcomes remain prudent.

In conclusion, this study represented a short- to mid-term follow-up non-registry review of the original and S + Attune TKR designs in comparison to its predicate design (PFC) as well as all other cemented implant designs used in a high-volume arthroplasty unit. There appeared to be no significantly increased revision rate for all-cause revision or aseptic loosening. Radiographic analysis also showed no significant difference in peri-implant radiolucency. It appears that concerns of early loosening may be unfounded.

## Data Availability

The datasets generated and/or analyzed during the current study are not publicly available as they contain potentially identifiable personal information but can be reviewed with the corresponding author on reasonable request and institutional consent.

## References

[1] No Authors Listed. NJR 19th Annual Report 2022. Available from: https://reports.njrcentre.org.uk/Portals/0/PDFdownloads/NJR%2019th%20Annual%20Report%202022.pdf. Accessed 11 Oct 2023.

[2] No Authors Listed. Australian Orthopaedic Association National Joint Replacement Registry: hip, knee & shoulder arthroplasty 2022 annual report. Available from: https://aoanjrr.sahmri.com/documents/10180/732916/AOA+2022+AR+Digital/f63ed890-36d0-c4b3-2e0b-7b63e2071b16. Accessed 11 Oct 2023.

[3] Kurtz S, Ong K, Lau E, Mowat F, Halpern M. Projections of primary and revision hip and knee arthroplasty in the United States from 2005 to 2030. J Bone Joint Surg Am. 2007;89(4):780–5. 17403800 10.2106/00004623-200704000-00012

[4] Sharkey PF, Lichstein PM, Shen C, Tokarski AT, Parvizi J. Why are total knee arthroplasties failing today–has anything changed after 10 years? J Arthroplasty. 2014;29(9):1774–8. 25007726 10.1016/j.arth.2013.07.024

[5] Lombardi AV, Berend KR, Adams JB. Why knee replacements fail in 2013: patient, surgeon, or implant? Bone Jt J. 2014;96-B(11 Supple A):101–4. 10.1302/0301-620X.96B11.34350 25381419

[6] Yang J, Heckmann ND, Nahhas CR, Salzano MB, Ruzich GP, Jacobs JJ, et al. Early outcomes of a modern cemented total knee arthroplasty : is tibial loosening a concern? Bone Jt J. 2021;103-B(6 Supple A):51–8. 10.1302/0301-620X.103B6.BJJ-2020-1972.R1 34053274

[7] Jaeger S, Eissler M, Schwarze M, Schonhoff M, Kretzer JP, Bitsch RG. Does tibial design modification improve implant stability for total knee arthroplasty? An experimental cadaver study. Bone Jt Res. 2022;11(4):229–38. 10.1302/2046-3758.114.BJR-2021-0169.R1 PMC9057524 35400170

[8] Bonutti PM, Khlopas A, Chughtai M, Cole C, Gwam CU, Harwin SF, et al. Unusually high rate of early failure of tibial component in ATTUNE total knee arthroplasty system at implant-cement interface. J Knee Surg. 2017;30(5):435–9. 28591930 10.1055/s-0037-1603756

[9] Scott CEH, Biant LC. The role of the design of tibial components and stems in knee replacement. J Bone Joint Surg Br. 2012;94(8):1009–15. 22844039 10.1302/0301-620X.94B8.28289

[10] Pittman GT, Peters CL, Hines JL, Bachus KN. Mechanical bond strength of the cement-tibial component interface in total knee arthroplasty. J Arthroplasty. 2006;21(6):883–8. 16950044 10.1016/j.arth.2005.10.006

[11] Kutzner I, Hallan G, Høl PJ, Furnes O, Gøthesen Ø, Figved W, et al. Early aseptic loosening of a mobile-bearing total knee replacement. Acta Orthop. 2018;89(1):77–83. 29105532 10.1080/17453674.2017.1398012 PMC5810837

[12] Willburger RE, Oberberg S. Early and mid-term results with the ATTUNE total knee replacement system compared to PFC Sigma: a prospective comparative study. J Orthop Surg. 2022;17(1):509. 10.1186/s13018-022-03397-7 PMC9694569 36434699

[13] Torino D, Damsgaard C, Kolessar DJ, Hayes DS, Foster B, Constantino J, et al. Tibial baseplate-cement interface debonding in the ATTUNE total knee arthroplasty system. Arthroplasty Today. 2022;17:165–71. 36164312 10.1016/j.artd.2022.06.012 PMC9508148

[14] Ranawat CS, White PB, West S, Ranawat AS. Clinical and radiographic results of attune and PFC sigma knee designs at 2-year follow-up: a prospective matched-pair analysis. J Arthroplasty. 2017;32(2):431–6. 27600300 10.1016/j.arth.2016.07.021

[15] Murphy JD, Braunlich PR, Judson Iv WR, Harker JN, Baumann PA. Early aseptic failure of the tibial component-cement interface in ATTUNE® total knee arthroplasty: a report of three cases. Cureus. 2021;13(12):e20582. 35103160 10.7759/cureus.20582 PMC8776517

[16] Cerquiglini A, Henckel J, Hothi H, Allen P, Lewis J, Eskelinen A, et al. Analysis of the Attune tibial tray backside: a comparative retrieval study. Bone Jt Res. 2019;8(3):136–45. 10.1302/2046-3758.83.BJJ-2018-0102.R2 PMC6446526 30997039

[17] Meneghini RM, Mont MA, Backstein DB, Bourne RB, Dennis DA, Scuderi GR. Development of a modern knee society radiographic evaluation system and methodology for total knee arthroplasty. J Arthroplasty. 2015;30(12):2311–4. 26122112 10.1016/j.arth.2015.05.049

[18] Refsum AM, Nguyen UV, Gjertsen JE, Espehaug B, Fenstad AM, Lein RK, et al. Cementing technique for primary knee arthroplasty: a scoping review. Acta Orthop. 2019;90(6):582–9. 31452416 10.1080/17453674.2019.1657333 PMC6844414

[19] van Otten TJM, van Loon CJM. Early aseptic loosening of the tibial component at the cement-implant interface in total knee arthroplasty: a narrative overview of potentially associated factors. Acta Orthop Belg. 2022;88(1):103–11. 35512160 10.52628/88.1.13

[20] Hoskins W, Rainbird S, Lorimer M, Graves SE, Bingham R. What can we learn from surgeons who perform THA and TKA and have the lowest revision rates? A study from the Australian orthopaedic association national joint replacement registry. Clin Orthop. 2022;480(3):464–81. 34677162 10.1097/CORR.0000000000002007 PMC8846272

[21] Billi F, Kavanaugh A, Schmalzried H, Schmalzried TP. Techniques for improving the initial strength of the tibial tray-cement interface bond. Bone Jt J. 2019;101-B(1_Supple_A):53–8. 10.1302/0301-620X.101B1.BJJ-2018-0500.R1 30648489

[22] Cox ZC, Engstrom SM, Shinar AA, Polkowski GG, Mason JB, Martin JR. Is cement mantle thickness a primary cause of aseptic tibial loosening following primary total knee arthroplasty? Knee. 2023;40:305–12. 36592499 10.1016/j.knee.2022.12.003

[23] Kopinski JE, Aggarwal A, Nunley RM, Barrack RL, Nam D. Failure at the tibial cement-implant interface with the use of high-viscosity cement in total knee arthroplasty. J Arthroplasty. 2016;31(11):2579–82. 27155996 10.1016/j.arth.2016.03.063

[24] Arsoy D, Pagnano MW, Lewallen DG, Hanssen AD, Sierra RJ. Aseptic tibial debonding as a cause of early failure in a modern total knee arthroplasty design. Clin Orthop. 2013;471(1):94–101. 22790529 10.1007/s11999-012-2467-4 PMC3528903

[25] Keohane D, Sheridan GA, Masterson E. High rate of tibial debonding and failure in a popular knee replacement. Bone Jt Open. 2022;3(6):495–501. 35698801 10.1302/2633-1462.36.BJO-2022-0043.R1 PMC9233423

[26] Lionberger D, Wattenbarger L, Conlon C, Walker TJ. Factors affecting aseptic loosening in primary total knee replacements: an in vitro study. J Exp Orthop. 2020;5(7):41. 10.1186/s40634-020-00243-9 PMC7275102 32504155

[27] Hazelwood KJ, O’Rourke M, Stamos VP, McMillan RD, Beigler D, Robb WJ. Case series report: early cement–implant interface fixation failure in total knee replacement. Knee. 2015;22(5):424–8. 25795544 10.1016/j.knee.2015.02.016

[28] Castagnini F, Sudanese A, Bordini B, Tassinari E, Stea S, Toni A. Total knee replacement in young patients: survival and causes of revision in a registry population. J Arthroplasty. 2017;32(11):3368–72. 28655567 10.1016/j.arth.2017.05.052

[29] Abdel MP, Bonadurer GF, Jennings MT, Hanssen AD. Increased aseptic tibial failures in patients with a BMI ≥35 and well-aligned total knee arthroplasties. J Arthroplasty. 2015;30(12):2181–4. 26220103 10.1016/j.arth.2015.06.057

[30] Gunst S, Fessy MH. The effect of obesity on mechanical failure after total knee arthroplasty. Ann Transl Med. 2015;3(20):310. 26697470 10.3978/j.issn.2305-5839.2015.10.37 PMC4669315

[31] Elcock KL, MacDonald DJ, Clement ND, Scott CEH. Total knee arthroplasty in patients with severe obesity: outcomes of standard keeled tibial components versus stemmed universal base plates. Knee Surg Relat Res. 2023;35(1):9. 37041576 10.1186/s43019-023-00184-4 PMC10088243

[32] van Loon C, Baas N, Huey V, Lesko J, Meermans G, Vergroesen D. Early outcomes and predictors of patient satisfaction after TKA: a prospective study of 200 cases with a contemporary cemented rotating platform implant design. J Exp Orthop. 2021;8(1):30. 33864173 10.1186/s40634-021-00347-w PMC8052397

[33] Lachiewicz PF, Steele JR, Wellman SS. Unexpected high rate of revision of a modern cemented fixed bearing modular posterior-stabilized knee arthroplasty. Bone Jt J. 2021;103-B(6 Supple A):137–44. 10.1302/0301-620X.103B6.BJJ-2020-1956.R1 34053294

[34] Robinson T, King SW, Pilling RWD, Aderinto J, Veysi V, Wall O, et al. Attune total knee arthroplasty: is there evidence of early tibial component de-bonding? A prospective cohort study with a minimum two year follow-up. J Arthrosc Jt Surg. 2021;8(2):139–47. 10.1016/j.jajs.2021.03.007

[35] Song SJ, Lee HW, Kang SG, Bae DK, Park CH. Various types of medial tibial bone resorption after total knee arthroplasty using a thick cobalt chromium tibial baseplate. J Knee Surg. 2022;35(4):434–42. 32838461 10.1055/s-0040-1715085

[36] Jin QH, Lee WG, Song EK, Kim WJ, Jin C, Seon JK. No difference in the anteroposterior stability between the GRADIUS and multi-radius designs in total knee arthroplasty. Knee. 2020;27(4):1197–204. 32711882 10.1016/j.knee.2020.05.019

[37] Kaptein BL, den Hollander P, Thomassen B, Fiocco M, Nelissen RGHH. A randomized controlled trial comparing tibial migration of the ATTUNE cemented cruciate-retaining knee prosthesis with the PFC-sigma design. Bone Jt J. 2020;102-B(9):1158–66. 10.1302/0301-620X.102B9.BJJ-2020-0096.R1 PMC7468556 32862688

[38] Hoskins W, Gorup P, Claireaux H, Stokes C, Bingham R. High incidence of radiolucent lines at the implant-cement interface of a new total knee replacement. ANZ J Surg. 2020;90(7–8):1299–302. 32536016 10.1111/ans.16046

[39] Staats K, Wannmacher T, Weihs V, Koller U, Kubista B, Windhager R. Modern cemented total knee arthroplasty design shows a higher incidence of radiolucent lines compared to its predecessor. Knee Surg Sports Traumatol Arthrosc Off J ESSKA. 2019;27(4):1148–55. 10.1007/s00167-018-5130-0 PMC6435629 30244340

[40] Bloch BV, Palan J, Shahid M, James PJ. A new total knee arthroplasty design has significantly better early implant survivorship than a previous gold-standard design-A retrospective analysis of 1,000 cases. J Knee Surg. 2020;33(2):152–7. 30708383 10.1055/s-0038-1676770

[41] Song SJ, Lee HW, Kang SG, Bae DK, Park CH. Various types of medial tibial bone resorption after total knee arthroplasty using a thick cobalt chromium tibial baseplate. J Knee Surg. 2022;35(4):434–42. 32838461 10.1055/s-0040-1715085

[42] Behrend H, Hochreiter B, Potocnik P, El Baz Y, Zdravkovic V, Tomazi T. No difference in radiolucent lines after TKA: a matched-pair analysis of the classic implant and its evolutional design. Knee Surg Sports Traumatol Arthrosc Off J ESSKA. 2020;28(12):3962–8. 10.1007/s00167-020-05894-w 32062683

[43] Giaretta S, Berti M, Micheloni GM, Ceccato A, Marangoni F, Momoli A. Early experience with the ATTUNE total knee replacement system. Acta Bio-Medica Atenei Parm. 2019;90(12-S):98–103. 10.23750/abm.v90i12-S.8997 PMC7233713 31821292

[44] Turgeon TR, Gascoyne TC, Laende EK, Dunbar MJ, Bohm ER, Richardson CG. The assessment of the stability of the tibial component of a novel knee arthroplasty system using radiostereometric analysis. Bone Jt J. 2018;100-B(12):1579–84. 10.1302/0301-620X.100B12.BJJ-2018-0566.R1 30499327

[45] Prodromidis AD, Chloros GD, Thivaios GC, Sutton PM, Pandit H, Giannoudis PV, et al. High rate of radiolucent lines following the cemented original design of the ATTUNE total knee arthroplasty. Bone Jt J. 2023;105-B(6):610–21. 10.1302/0301-620X.105B6.BJJ-2022-0675.R1 37259548

[46] Chalmers BP, Sculco PK, Fehring KA, Taunton MJ, Trousdale RT. Fluoroscopically assisted radiographs improve sensitivity of detecting loose tibial implants in revision total knee arthroplasty. J Arthroplasty. 2017;32(2):570–4. 27665244 10.1016/j.arth.2016.08.005

[47] Bayliss LE, Culliford D, Monk AP, Glyn-Jones S, Prieto-Alhambra D, Judge A, et al. The effect of patient age at intervention on risk of implant revision after total replacement of the hip or knee: a population-based cohort study. Lancet Lond Engl. 2017;389(10077):1424–30. 10.1016/S0140-6736(17)30059-4 PMC5522532 28209371

[48] Meehan JP, Danielsen B, Kim SH, Jamali AA, White RH. Younger age is associated with a higher risk of early periprosthetic joint infection and aseptic mechanical failure after total knee arthroplasty. J Bone Joint Surg Am. 2014;96(7):529–35. 24695918 10.2106/JBJS.M.00545

[49] Aggarwal VK, Goyal N, Deirmengian G, Rangavajulla A, Parvizi J, Austin MS. Revision total knee arthroplasty in the young patient: is there trouble on the horizon? J Bone Joint Surg Am. 2014;96(7):536–42. 24695919 10.2106/JBJS.M.00131

[50] Matar HE, Platt SR, Gollish JD, Cameron HU. Overview of randomized controlled trials in total knee arthroplasty (47,675 Patients): what have we learnt? J Arthroplasty. 2020;35(6):1729-1736.e1. 32088054 10.1016/j.arth.2020.01.065

[51] Clark CR, Rorabeck CH, MacDonald S, MacDonald D, Swafford J, Cleland D. Posterior-stabilized and cruciate-retaining total knee replacement: a randomized study. Clin Orthop. 2001;392:208–12. 10.1097/00003086-200111000-00025 11716384

[52] Luo SX, Zhao JM, Su W, Li XF, Dong GF. Posterior cruciate substituting versus posterior cruciate retaining total knee arthroplasty prostheses: a meta-analysis. Knee. 2012;19(4):246–52. 22300844 10.1016/j.knee.2011.12.005

[53] Nouta KA, Verra WC, Pijls BG, Schoones JW, Nelissen RGHH. All-polyethylene tibial components are equal to metal-backed components: systematic review and meta-regression. Clin Orthop. 2012;470(12):3549–59. 22972656 10.1007/s11999-012-2582-2 PMC3492632

[54] Wang K, Zhang FF, Yan X, Shen Y, Cai W, Xu J, et al. Superior mid- to long-term clinical outcomes of mobile-bearing total knee arthroplasty compared to fixed-bearing: a meta-analysis based on a minimum of 5 years of study. J Knee Surg. 2021;34(12):1368–78. 32503063 10.1055/s-0040-1709490

[55] Powell AJ, Crua E, Chong BC, Gordon R, McAuslan A, Pitto RP, et al. A randomized prospective study comparing mobile-bearing against fixed-bearing PFC Sigma cruciate-retaining total knee arthroplasties with ten-year minimum follow-up. Bone Jt J. 2018;100-B(10):1336–44. 10.1302/0301-620X.100B10.BJJ-2017-1450.R1 30295539

[56] Killen CJ, Murphy MP, Hopkinson WJ, Harrington MA, Adams WH, Rees HW. Minimum twelve-year follow-up of fixed- vs mobile-bearing total knee arthroplasty: double blinded randomized trial. J Clin Orthop Trauma. 2020;11(1):154–9. 32002005 10.1016/j.jcot.2019.03.019 PMC6985168

[57] Price AJ, Rees JL, Beard D, Juszczak E, Carter S, White S, et al. A mobile-bearing total knee prosthesis compared with a fixed-bearing prosthesis. A multicentre single-blind randomised controlled trial. J Bone Joint Surg Br. 2003;85(1):62–7. 12585579 10.1302/0301-620X.85B1.13233

